# Self-Assembled Synthesis of Porous Iron-Doped Graphitic Carbon Nitride Nanostructures for Efficient Photocatalytic Hydrogen Evolution and Nitrogen Fixation

**DOI:** 10.3390/nano13020275

**Published:** 2023-01-09

**Authors:** Valmiki B. Koli, Gavaskar Murugan, Shyue-Chu Ke

**Affiliations:** Department of Physics, National Dong Hwa University Shou-Feng, Hualien 97401, Taiwan

**Keywords:** Fe-doped MCNC, supramolecular approach, thermal exfoliation, visible-light-active, photocatalytic hydrogen evolution, nitrogen fixation

## Abstract

In this study, Fe-doped graphitic carbon nitride (Fe-MCNC) with varying Fe contents was synthesized via a supramolecular approach, followed by thermal exfoliation, and was then used for accelerated photocatalytic hydrogen evolution and nitrogen fixation. Various techniques were used to study the physicochemical properties of the MCN (g-C_3_N_4_ from melamine) and Fe-MCNC (MCN for g-C_3_N_4_ and C for cyanuric acid) catalysts. The field emission scanning electron microscopy (FE-SEM) images clearly demonstrate that the morphology of Fe-MCNC changes from planar sheets to porous, partially twisted (partially developed nanotube and nanorod) nanostructures. The elemental mapping study confirms the uniform distribution of Fe on the MCNC surface. The X-ray photoelectron spectroscopy (XPS) and UV-visible diffuse reflectance spectroscopy (UV-DRS) results suggest that the Fe species might exist in the Fe^3+^ state and form Fe-N bonds with N atoms, thereby extending the visible light absorption areas and decreasing the band gap of MCN. Furthermore, doping with precise amounts of Fe might induce exfoliation and increase the specific surface area, but excessive Fe could destroy the MCN structure. The optimized Fe-MCNC nanostructure had a specific surface area of 23.6 m^2^ g^−1^, which was 8.1 times greater than that of MCN (2.89 m^2^ g^−1^). To study its photocatalytic properties, the nanostructure was tested for photocatalytic hydrogen evolution and nitrogen fixation; 2Fe-MCNC shows the highest photocatalytic activity, which is approximately 13.3 times and 2.4 times better, respectively, than MCN-1H. Due to its high efficiency and stability, the Fe-MCNC nanostructure is a promising and ideal photocatalyst for a wide range of applications.

## 1. Introduction

In 2009, graphite-phase g-C_3_N_4_ (MCN) with earth-abundant elements (C and N) was used as a metal-free photocatalyst [1]. This has since become a popular research topic due to its appealing visible-light response, moderate bandgap (2.7 eV), excellent stability, and low cost. Therefore, this is one of the most promising new visible-light-responsive photocatalysts for various applications, such as hydrogen evolution [2], carbon dioxide reduction [3], nitrogen fixation [4], hydrogen peroxide production [5], and environmental remediation [6]. However, it also suffers from a few limitations, such as low solar energy conversion efficiency due to the high recombination rate of the photogenerated electron hole, and a narrow visible-light response (460 nm) [7]. The solar spectrum contains 45% visible light and 50% near-infrared light. Broadening the photocatalyst’s light-response range and lowering the photogenerated carrier’s recombination rate are critical for efficient solar energy conversion. To overcome this limitation, researchers applied various strategies, such as doping with metal or non-metal [8,9], making hybrid nanocomposites with metal oxides and carbon nanostructures [10,11], loading noble metal [12], changing the morphology [13,14], and chemical and thermal exfoliation [15]. All these strategies improved the photocatalytic activity of MCN. However, the photocatalytic activity was not yet up to the requisite standard. Therefore, researchers applied other strategies, such as a co-doping of metal and non-metal [16], making binary ternary quaternary hybrid composites [17], and multistep heat treatment [18], all of which showed improvement in the photocatalytic properties of MCN.

As a result, combining multiple strategies to improve photocatalytic efficiency may represent a novel approach to the synthesis of highly porous visible-light-active photocatalysts. Therefore, in the present work, we applied three different strategies to synthesize porous Fe-doped MCN nanostructures. First, the strategy of Fe-doping in MCN has long been considered an efficient and straightforward method of modifying MCN because Fe is a more familiar, less toxic, and naturally abundant element. Specifically, MCN contains six lone-pair nitrogen electrons in the form of “nitrogen pots.” This one-of-a-kind framework lends itself perfectly to Fe integration. Many researchers found that adding low-concentration Fe species to MCN significantly improved its electronic properties as well as the catalyst’s performance [19]. In a Fe-doped MCN system, Fe can exist in Fe–CN, in the form of Fe–N ligands. Under visible-light illumination, photoinduced electrons from Fe–CN can also reduce Fe^3+^ to Fe^2+^. As a result, Fe–CN can be used as a catalyst in a heterogeneous photocatalytic reaction [20]. The second strategy is a change in the morphology, using the supramolecular approach for the synthesis of porous MCN. This is a typical physicochemical feature, with enlarged surface areas, promoted photoinduced charge separation, and more reactive sites being produced. A quick, secure, and affordable method for fabricating porous MCN is by self-templating supramolecular melamine with cyanuric acid [21,22,23]. A third strategy, the thermal oxidation (exfoliation) of bulk MCN, has been used to create porous MCN materials [24,25]. Appropriate templates can simply alter the porosity, structure, morphology, surface area, and size. The remarkable structures and high porosity of the MCN used in this study can result in enormous surface areas and a large number of active sites for diverse application-driven photocatalysis.

Thermal exfoliation can yield high-quality free-standing layers of MCN nanosheets with large specific surface areas, increased charge transfer and separation efficiency, and a lower charge-carrier recombination rate. The use of templating and etching agents, on the other hand, is not environmentally friendly. The photocatalytic activity of synthesized porous Fe-MCNC was tested against photocatalytic hydrogen evolution and N_2_ fixation because today’s excessive consumption and extraction of fossil fuels, energy shortages, and environmental pollution have suddenly emerged as significant concerns that endanger society’s survival and development [26]. As a result, renewable and green energy must be sought to replace these fossil fuels. Hydrogen and ammonia have been viewed as green energy sources since they emit no CO_2_ and have a high energy density [27,28]. All characterization data and photocatalytic studies show that 2Fe-MCNC is the most effective photocatalyst. It has a 13.3-fold higher efficiency than MCN-1H in photocatalytic hydrogen evolution; a similar trend was observed in N_2_ fixation, where 2Fe-MCNC has a 2.4-fold higher NH_4_^+^ concentration than MCN-1H. Based on these findings, a photocatalytic hydrogen evolution and nitrogen fixation mechanism has been proposed.

## 2. Materials and Methods

### 2.1. Chemicals

Melamine, cyanuric acid (purchased from Alfa Aesar, UK), chloroplatinic acid hexahydrate, ethanol, methanol, and ferric nitrate nonahydrate were purchased from Thermo Scientific (Waltham, MA, USA). Nessler’s reagent was prepared by mixing 2 g of potassium iodide in 5 mL water. To this solution, 3 g of mercury (II) iodide was added, and the resulting solution was made up to 20 mL. Finally, 40 g potassium hydroxide (30%) was added to provide the alkaline base. All reagents were purchased from Sigma Aldrich, (Burlington, MA, USA).

#### 2.1.1. Synthesis of Pure g-C_3_N_4_ (MCN)

The pristine nanostructured MCN sample was synthesized by heating 5 gm of melamine at 520 °C for 4 h in a box furnace with a heating rate of 5 °C/min. The sample was then designated as MCN-1H. For thermal exfoliation, the same sample was heated at 600 °C for 2 h in a tube furnace under nitrogen flow with a heating rate of 5 °C/min, and this sample was designated as MCN-2H.

#### 2.1.2. Synthesis of Fe-MCNC with Supramolecular Approach

To begin, a Fe stock solution was prepared by dispersing 100 mg ferric nitrate nonahydrate in 50 mL of distilled water. The melamine–cyanuric acid complexes were created using a slightly modified technique to that described in an earlier work [29]. In brief, equimolar volumes of cyanuric acid and melamine were completely mixed in distilled water for 30 min before adding a stochiometric amount of ferric nitrate nonahydrate solution (0.5, 1, 2, 3, and 4 mL) and then stirred for 6 h at room temperature, to form supramolecular adducts. The complex was then filtered using Whatman filter paper and dried at 70 °C overnight. The resulting supramolecular adducts were ground up and heated in a box furnace at 520 °C for 4 h at 5 °C/min. They were then allowed to cool before grinding again and heat-treating in a tube furnace with nitrogen flow at 600 °C for 2 h at 5 °C/min. The synthesized samples were denoted by the quantity of Fe solutions in the sample, as 0.5Fe-MCNC, 1Fe-MCNC, 2Fe-MCNC, 3Fe-MCNC, and 4Fe-MCNC (where “Fe” represents iron, “MCN” represents the g-C_3_N_4_ from melamine, and “C” represents cyanuric acid). A similar technique was utilized to synthesize MCNC without the inclusion of Fe solutions.

### 2.2. Photocatalytic Hydrogen Evolution

Photocatalytic hydrogen evolution was performed to test the photocatalytic activity of a synthesized catalyst under visible light, using a 350-W Xenon lamp and an optical cut-off filter (>420 nm). The entire system was evacuated after dispersing 25 mg of photocatalyst powder in 50 mL of a 10% triethanolamine aqueous solution and a 1.0 wt % H_2_PtCl_6_ solution. The Pt nanoparticles were deposited on the catalyst surface by photo-deposition at 25 °C for 30 min, under UV-Vis light irradiation with steady stirring. The reaction chamber was then irradiated with only visible light through a UV cut-off filter, then the generated H_2_ was measured every 30 min with an auto-sampler connected to a gas chromatography device (GC-2030).

### 2.3. Photocatalytic Nitrogen Fixation Experiments

The photocatalytic N_2_ fixing experiments were carried out in a 100 mL reaction cell at a constant temperature and pressure. In a typical experiment, 20 mg of photocatalyst powder was suspended in 50 mL of a 20% aqueous methanol solution, and pure nitrogen gas was bubbled through for 1h. Then, the reactor was sealed and exposed to white light from a 65 W LED bulb. Following that, 2 mL of the suspension was withdrawn from the reaction cell during the light irradiation for NH_4_^+^ concentration analysis. Nessler’s reagent method was used to determine the NH_4_^+^ concentration. The suspension was filtered through a 0.42-µm membrane filter before being poured into a 2.5-mL sample tube. Following that, 100 µL of potassium tartrate and 100 µL of Nessler’s reagent were added, and the mixture was left to stand for 10 min in order to assess the color change. Finally, the UV-Vis spectrophotometer (Shimadzu UV-2550) was used to measure the NH_4_^+^ concentration by recording the absorbance at 430 nm.

### 2.4. Electrochemical Analysis

In 1.0 mL of ethanol, 1.0 mg of the catalyst was ultrasonically combined with 50 µL of Nafion solution (acting as a binder). The suspension was deposited on a glassy carbon electrode and allowed to dry at room temperature for 20 min. This procedure was performed three times to achieve consistent catalyst deposition on the glassy carbon electrode. In a three-electrode system with a true contact, the Pt electrode, Ag/AgCl/KCl (sat) electrode, and fabricated glassy carbon electrode were employed as the counter, reference, and working electrodes. An electrochemical workstation (Metrohm Autolab PGSTA204 with an FRA32M module (Metrohm, Utrecht, Netherlands) was utilized to collect and analyze the data from the photocurrent response, electrochemical impedance, and Mott–Schottky tests, performed with a 0.1 mM Na_2_SO_4_ solution (pH 7.0).

### 2.5. Characterization

The morphology and elemental contents of the representative samples were examined using field emission scanning electron microscopy (FE-SEM model: JSM 7000F, JEOL Ltd., Tokyo, Japan) and energy-dispersive X-ray spectrometry (EDS model: JEOL Ltd., Tokyo, Japan). The phase and crystal structures of the synthesized nanomaterials were determined by X-ray diffraction analysis (XRD model: Bruker, D2 Phaser Benchtop XRD Germany) with Cu K radiation (=1.54056). The UV-visible diffuse reflectance spectra (UV-Vis DRS model: UV-2550 UV-visible spectrophotometer Shimadzu Japan) were used to examine the optical properties. Various functional moieties on the surface of a manufactured catalyst were investigated using Fourier transform infrared analysis (FTIR model: Perkin Elmer, Waltham, MA, USA) in the 400–4000 cm^−1^ wavenumber range. The samples for the FT-IR investigations were prepared using the standard KBr pellet preparation procedure. To accomplish the X-ray photoelectron spectroscopy observations (XPS model: Thermo Scientific, Waltham, MA, USA), monochromatic Al K radiation and a charge neutralizer were used. Nitrogen adsorption-desorption experiments were carried out at 77 K, utilizing the Quantachrome Autosorb (Quantachrome, Boynton Beach, FL, USA). X-band electron paramagnetic resonance (EPR) spectra were collected at 77 K using a Bruker EMX spectrometer (Bruker, Karlsruhe, Germany). An electrochemical workstation was used to investigate the electrochemical properties, fitted with a Metrohm Autolab PGSTA204 with an FRA32M module (Metrohm, Utrecht, Netherlands). Gas chromatography was used to examine hydrogen generation (GC model: Nexis GC-2030, Japan).

## 3. Results and Discussion

**Figure 1:**
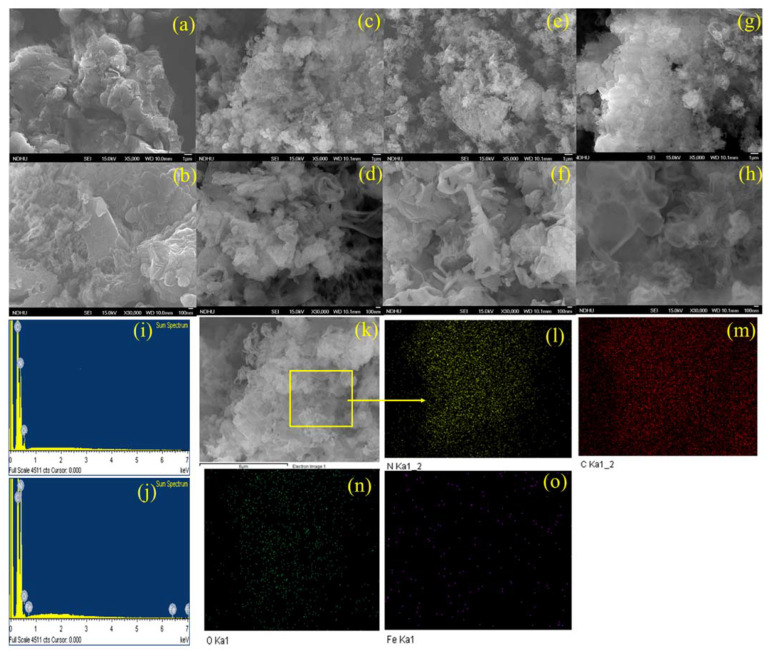
FE-SEM images: (**a**,**b**) MCN-1H, (**c**,**d**) 1Fe-MCNC, (**e**,**f**) 2Fe-MCNC, (**g**,**h**) 3Fe-MCNC, (**i**,**j**) EDS spectra of MCN-1H and 2Fe-MCNC, and (**k**–**o**) FE-SEM image and elemental mapping of 2Fe-MCNC.

The microstructures and surface morphologies of the catalysts were studied using FE-SEM at two different magnifications. As illustrated in Figure 1a,b, MCN-1H is a flat-shaped sheet with uneven lamellar stacking and no apparent pore structure. It has a typical bulk-like morphology, with very compact agglomerates of carbon nitride sheets that appear as scales protruding from the material. This characteristic can help explain the low specific surface area [30]. The dense, layered structure of the MCN catalyst started to break down after Fe doping, forming a porous structure due to the generation of a considerable number of gaseous chemicals, such as CO_2_, SO_2_, NO_x_, H_2_S, or NH_3_, during the high-temperature pyrolysis process, as shown in Figure 1c–h. This suggested that Fe doping could modify MCN, while also increasing the pore volume of the catalyst. The Fe-MCNC photocatalyst has an entirely different form. It demonstrates the synthesis of homogeneous and highly porous partially formed nanotube and nanorod-shaped Fe-MCNC, using a supramolecular approach with a melamine and cyanuric acid mixture [31]. In addition, there are obvious flat and layered structures, and the material is composed of thin carbon nitride sheets that have been partially twisted in on themselves, as seen in Figure 1c–f. Because of the presence of cyanuric acid and Fe doping during synthesis, the completely exfoliated morphology of this material can also justify its higher specific surface area when compared to MCN-1H. In Figure 1g–h, the sheets appear to be more transparent and with reduced thickness, implying a reduction in the number of layers in the stacking when compared to MCN-1H and 1- and 2Fe-MCNC. This means that an excess of Fe doping promotes the exfoliation of the stacked layers of MCN. The EDS spectra of MCN-1H and 2Fe-MCNC confirm the Fe doping in the MCNC nanostructure (see Figure 1i,j). The elemental mapping images in Figure 1l–o revealed a uniform distribution of C, N, O, and Fe elements in the framework of the 2Fe-MCNC structure, showing that Fe was successfully incorporated into the structure. Table 1 depicts the elemental composition with the wt % values of representative MCN and Fe-MCNC nanostructures.

**Figure 2:**
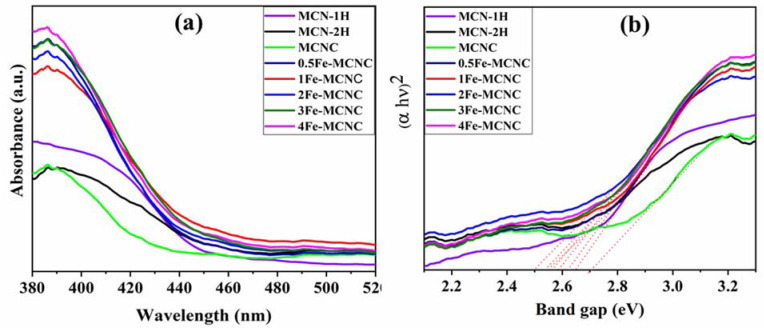
(**a**) UV-Vis DRS spectra and (**b**) Tauc plot of the MCN and Fe-MCNC nanostructures. (Red dotted lines are the tangents, drawn here to represent the band gap values of the samples).

The optical properties of synthetic nanomaterials were further investigated via UV-Vis DRS, as shown in Figure 2a,b. The absorbance spectra demonstrate that the Fe-MCNC exhibits red shifts when compared to MCN-1H, corresponding to a band gap decrease from 2.70 eV to 2.50 eV. This effect is observed in MCN-2H and Fe-MCNC, due to thermal exfoliation and doped Fe in the MCNC lattice, which results in a deformed band structure; hence, electrons in the Fe-MCNC can easily shuttle between the conduction (CB) and valence bands (VB) [32]. However, Fe doping decreased the band gap and shifted the absorption edge toward the visible region. With the addition of Fe dopants, the absorption edge of the Fe-MCNC redshifts to around 430–460 nm, as seen in Figure 2a. It could be linked to the Fe-CN conjugation and charge transfer. Their absorption tails are also wider and stronger, which improves visible light absorption and photocatalytic activity. The Tauc plot, shown in Figure 2b, was used to determine the band gap value for all samples.

**Figure 3:**
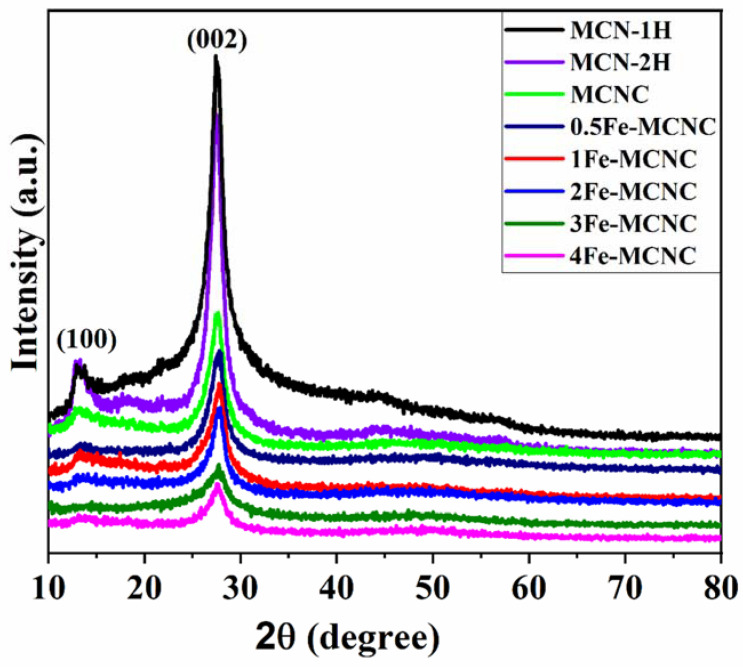
XRD patterns of the MCN and Fe-MCNC nanostructures.

The XRD patterns of MCN-1H, MCN-2H, and Fe-MCNC are shown in Figure 3. MCN-1H and MCN-2H had two unique diffraction peaks at around 13.07°and 27.54°, indicating that they shared the same crystal-structure matches (JCPDS No. 87-1526). The (002) and (100) crystal planes of g-C_3_N_4_ were responsible for these two peaks, which are associated with the inter-planar stacking peak of the heptazine units and the in-plane structural packing pattern of the tris-triazine units, respectively [33]. However, MCN-2H had weaker peaks than MCN-1H. This reveals the efficient exfoliation of the MCN-2H sample [34]. Compared to the XRD pattern of MCN-1H and MCN-2H, the (100) peak of MCNC was barely discernible, indicating that MCNC had a reduced planar dimension [35]. In the meantime, the intensity of the (002) peak decreased; the overall strength of the diffraction peaks of Fe-MCNC became weaker as the Fe content increased in the XRD patterns. It was revealed that Fe and MCNC showed host–guest interactions, in which a small amount of Fe species might inhibit MCNC condensation [19]. Interestingly, when the Fe content increased, the intensity of the (002) peak reduced significantly, indicating that too much Fe was unprofitable for MCNC exfoliation. Furthermore, despite the fact that the crystallinity of Fe-MCNC decreased as the Fe concentration increased, the MCN’s primary (002) peak was retained in Fe-MCNC. Therefore, it is shown that the Fe species was chemically connected to the MCNC, which served as a host, resulting in Fe–N connections. Following Fe doping, the (100) peak vanished, demonstrating the presence of Fe species embedded into the in-planes [36], which finding was remarkably analogous to those in previous metal-doping works on g-C_3_N_4_ materials [19,37]. Interestingly, no typical XRD peaks associated with iron-containing compounds were found in the Fe-MCNC samples, showing that the Fe element was successfully integrated into the framework of MCNC in a widely scattered condition, most likely via the Fe-N bonds [38]. According to some findings in the literature, the lack of a (100) peak was ascribed to restocking along (001) in the Fe-direction of MCNC [19].

**Figure 4:**
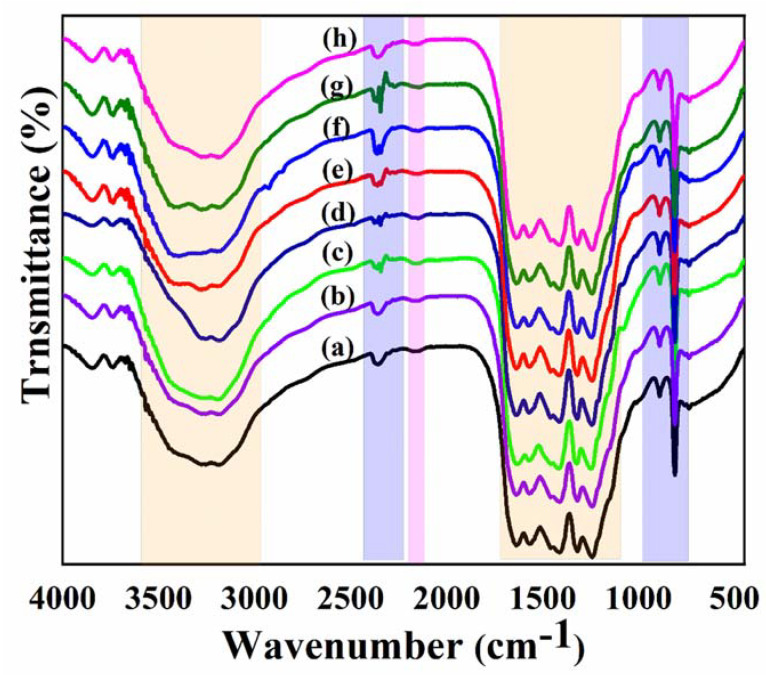
FTIR spectra of (a) MCN-1H, (b) MCN-2H, (c) MCNC, (d) 0.5 Fe-MCNC, (e) 1Fe-MCNC, (f) 2Fe-MCNC, (g) 3Fe-MCNC, and (h) 4Fe-MCNC.

Figure 4 depicts the FT-IR spectra of all synthesized samples. The broad absorption bands between 3600 and 3000 cm^−1^, in particular, were formed by N-H and O-H stretching vibrations, indicating that amino groups on the surface of the MCN and Fe-MCNC samples were only partially bonded together and that hydroxyl species had been attracted to the surface [39]. The CN heterocycle stretching vibrations were related to the strong band from 1700 to 1100 cm^−1^, with characteristic peaks at around 1643, 1561, 1408, 1324, and 1245 cm^−1^ [40], while the absorption band at 894 cm^−1^ corresponds to the N-H deformation mode from these groups [15]. Furthermore, the s-triazine ring system was linked to the sharp characteristic peak at 809 cm^−1^ [40]. Moreover, the catalyst showed an absorption peak at 2161 cm^−1^ that corresponded to one in CN, as illustrated in Figure 4. Furthermore, Fe-MCNC composites with varying doping amounts displayed comparable MCN absorption peaks, showing that the addition of Fe species had no effect on MCN’s chemical skeleton. The peak at 2355 cm^−1^ is induced by CO_2_ in the air, and this peak can be seen in both MCN and Fe-MCNC [41].

**Figure 5:**
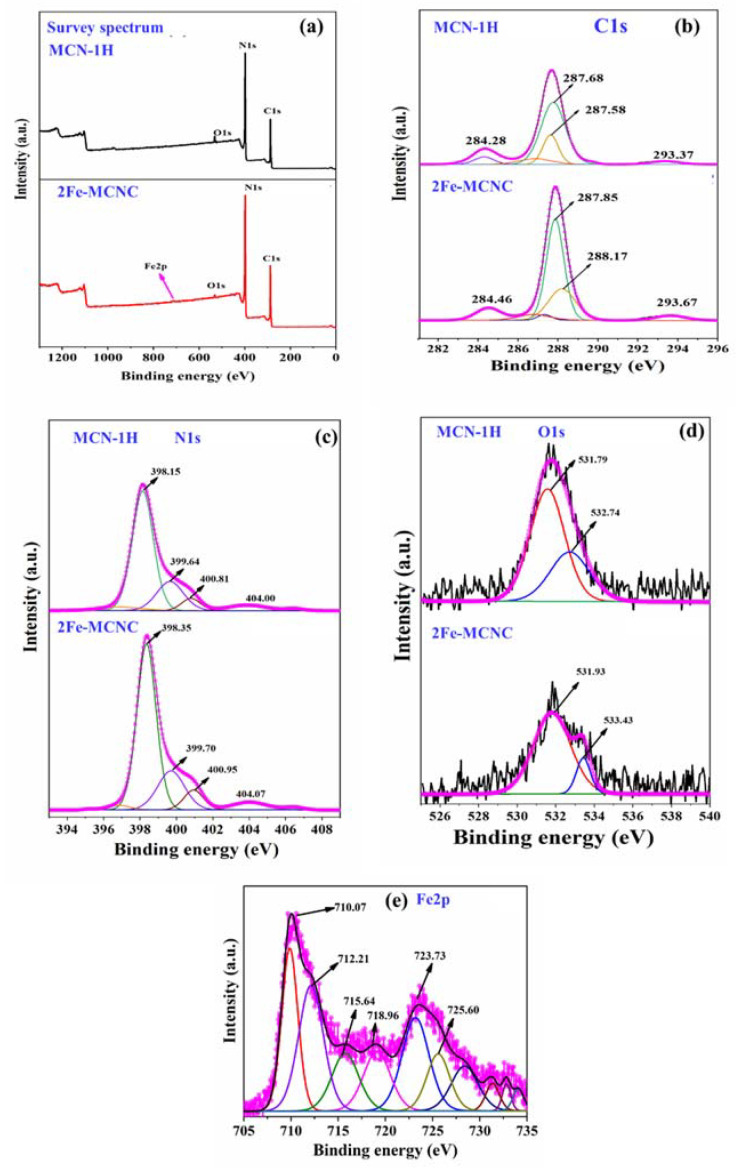
Comparative XPS spectra of MCN-1H and 2Fe-MCNC (**a**) survey spectrum, high-resolution XPS spectra (**b**) C1s, (**c**) N1s (**d**) O1s, and (**e**) Fe2p.

XPS was used to evaluate the elemental composition and chemical status of the representative MCN-1H and 2Fe-MCNC catalysts. Peaks of C, N, and O were observed in the MCN-1H XPS survey spectrum (see Figure 5a). The 2Fe-MCNC spectrum contained peaks of the C, N, O, and Fe elements. The most prevalent element was N, followed by C, O, and Fe. The adsorbed O_2_ and H_2_O produced trace amounts of oxygen [42]. Figure 5b–e illustrates the analysis of the specific peak data for C1s, N1s, O1s, and Fe2p. The high-resolution C1s spectra were fitted into three separate peaks, as seen in Figure 5b. The three peaks of MCN have binding energies of 284.28, 286.73, and 287.58 eV. The interfering carbon’s C-C bonds were associated with the peak at 284.91 eV. Peaks at 286.73 and 287.58 eV were ascribed to the C=N and (N)_2_-C=N groups, which arose from the tri-s-triazine unit that forms the MCN backbone [43]. The C1s spectra of 2Fe-MCNC were similar to that of MCN-1H, but with a higher proportion of C=N and (N)_2_-C=N groups, and their peaks were shifted to higher binding energies by 0.17 and 0.59 eV, respectively. These modifications could be attributed to the creation of Fe-Nx bonds during the Fe doping of MCNC, which influenced the chemical environment of the nearby C atoms [32].

Figure 5c depicts the high-resolution N1s spectra; the MCN-1H spectrum is separated into three peaks at 398.15, 399.64, and 400.81 eV, respectively. The intense peak at 398.15 eV has been attributed to SP^2^ N (C-N=C or pyridine N) in triazine units [44], whereas the peaks at 399.64 eV and 400.81 eV were attributed to the tertiary nitrogen (N-(C)3 or graphite N) and amino functional groups (C-N-H), respectively [44]. The high-resolution spectra of 2Fe-MCNC N1s revealed the same three distinct peaks. These N states are the fundamental unit of MCN, revealing that it is unaffected by Fe (III) species. However, the binding energies of these three peaks in 2Fe-MCNC changed somewhat from those in MCN-1H, indicating a change in the chemical state of hybridized N, as a result of Fe doping. Excitation or the charging effect in the cyano group and heterocycles could account for the small peak at 404.4 eV [19]. The high-resolution O1s spectra of these samples are shown in Figure 5d. The peak at 531.57 eV belongs to the N-CO species and the peaks at 532.74 eV are assigned to the absorbed H_2_O and O_2_ in MCN, while the same peak is present in 2Fe-MCNC, with slightly higher binding energies at 531.93 eV and 533.43 eV. There is a decrease in peak intensity in 2Fe-MCNC, suggesting that oxygen vacancies are present [45]. In terms of the Fe2p spectrum, Figure 5e depicts the Fe 2p XPS spectrum. The peaks at 710.07 and 723.7 eV were found to correspond to Fe 2p3/2 and Fe 2p1/2, respectively. The peak 710 eV binding energy was extremely close to previous findings [46], The peaks located at 710.07, 725.60, and 712.2 eV, 725.0 eV, which can be attributed to the Fe^2+^ and Fe^3+^. which revealed that the Fe species was stabilized in the electron-rich MCN structure via Fe-N bonds [47].

**Figure 6:**
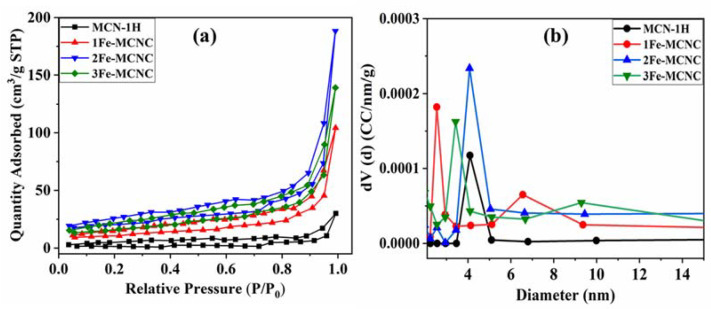
(**a**) The nitrogen adsorption–desorption BET isotherm, (**b**) pore size distribution of the MCN and Fe-MCNC nanostructures.

The pore size distribution of the low-temperature nitrogen adsorption method is typically calculated using the BJH method, which is based on the Kelvin equation (Equation (1)). The representative sample’s adsorption-desorption isotherms were of type IV, with an H3 hysteresis loop, confirming the presence of mesopores (see Figure 6a). The pore size distribution is calculated using the BJH method and is shown in Figure 6b. In the figure, the vertical coordinate dV/dD represents the pore volume versus the pore diameter differential. The surface area and pore size distribution of the catalyst are significant in the production of hydrogen, as shown in Table 2. The pore size distribution also shows that the average pore size of all samples is between 2.6 and 6.6 nm. Overall, 2Fe-MCNC had the highest pore segregation, with a size of 4.07 nm. However, it is clear that 1Fe-MCNC dominates the 2.55 nm pore size domain, 3Fe-MCNC rules the 3.4 nm region, and MCN-1H occupies the 4.1 nm regime with the smallest pore volume. Table 2 shows that the 2Fe-MCNC sample has the highest specific surface area, which is approximately 8.1 times that of MCN. The higher specific surface area not only provides an appropriate charge transport pathway but also offers more adsorption and reaction sites for the photocatalytic reaction process, enhancing photocatalytic hydrogen production [48]. As shown in Table 2, increasing the dopant concentration (3Fe-MCNC) reduces the specific surface area, which could be due to dopant agglomeration [49].

(1) $$ln\frac{P}{P_{0}}=-\frac{2\gamma \tilde{v}cos\theta }{rkRT}$$

Here, *P*/*P*_0_ is the equilibrium pressure over the saturated vapor pressure of the adsorbed gas; $\tilde{v}$ is the mole volume of the liquid nitrogen; *γ* is the interfacial tension of the liquid nitrogen.

**Figure 7:**
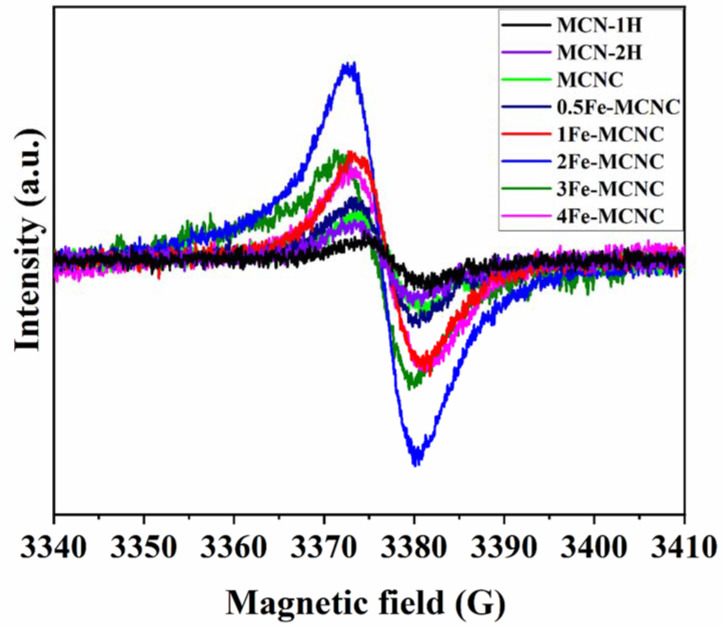
Visible-dark EPR spectra of MCN and Fe-MCNC at 77 K.

Figure 7 shows the visible minus dark (Vis-dark) EPR (electron paramagnetic resonance) spectra of the MCN and Fe-MCNC catalysts. Only one Lorentzian line, centered at 3380 G, with a g value of 2.004 is detected for unpaired electrons, generated in π-conjugated aromatic rings of carbon nitride [50]. When compared to MCN, Fe-MCNC samples have a higher EPR signal intensity, indicating that the inclusion of the benzene ring significantly extended the π-conjugated system and boosted electron delocalization, resulting in the observed increase in photocatalytic activity. The signal intensity of Fe-MCNC exceeds that of MCN, owing to the Fe doping and supramolecular approach, which increased the specific surface area and pore volume. The intensity of the EPR of Fe-MCNC increased under visible light irradiation, confirming the efficient production of photo-induced carrier pairs.

**Figure 8:**
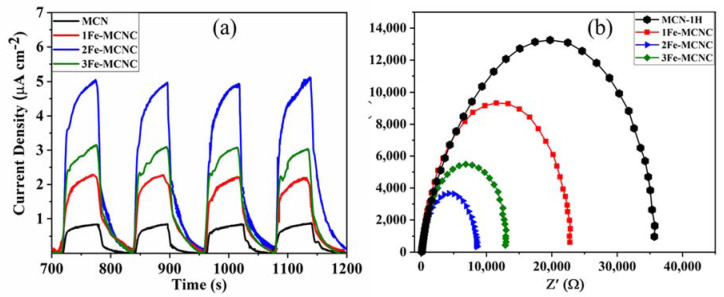
(**a**,**b**) Transient photocurrent responses and Nyquist plots of MCN and Fe-MCNC nanostructures.

The representative samples of transient photocurrent response are shown in Figure 8a; when the light is turned on, the photocurrent quickly rises to a higher value, and when the light is turned off, it falls back to its initial value. This revealed a quick and consistent photocurrent response that was repeatable for each on-off cycle. The current density of 2Fe-MCNC is five times more than that of MCN-1H, showing the most efficient separation and transition of photoinduced electron/hole pairs, which is consistent with UV DRS and catalytic activity. The Nyquist plots of the representative samples in Figure 8b show that Fe-MCNC has a lower arc radius because of the reduced electronic resistance and improved electronic conductivity generated by Fe doping, relative to MCN. The arc radius for 2Fe-MCNC is the shortest among the four samples, showing that the charge separation is the most efficient.

**Figure 9:**
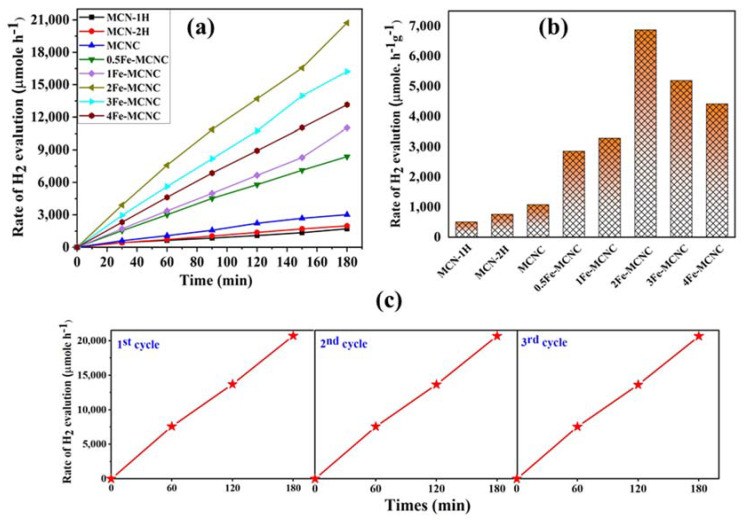
(**a**,**b**) The photocatalytic H_2_ evolution of the MCN and Fe-MCNC nanostructures, (**c**) the recycling stability experiments of 2Fe-MCNC for photocatalytic H_2_ evolution.

The photocatalytic performances of MCN and Fe-MCNC were studied via photocatalytic H_2_ generation, under visible-light irradiation from water in the presence of TEOA as a scavenger and Pt as a co-catalyst (see Figure 9a). The UV-Vis DRS spectra confirmed that the synthesized materials have a visible light response (Figure 2a) and the photocurrent studies revealed that MCN had a low photocurrent at the µA cm^−2^ scale (Figure 8a). The observation implied that the as-prepared MCN’s light sensitivity was quite poor. As a result, relatively low H_2_ production was found. Furthermore, it is widely known that using Pt nanoparticles as a co-catalyst can trap electrons, supply proton reduction sites, improve sluggish H_2_ production dynamics, and ultimately boost the H_2_ evolution efficiency. The intrinsically twisted Fe-MCNC nanostructure provides a framework for the insertion of a co-catalyst to further ramp up H_2_ generation. Figure 9a depicts a comparison of the photocatalytic H_2_ evolution rates with MCN and Fe-MCNC. The visible-light-driven H_2_ production of MCN is very inefficient with loading of 1% Pt nanoparticles. As expected, 2Fe-MCNC demonstrated significantly higher photocatalytic H_2_-evolved activity than MCN under the same conditions. The rate of H_2_-evolution for 2Fe-MCNC is 6880 µmol/h/g, which was approximately 13.30-fold higher than MCN-1H (517 µmol/h/g). It should be noted that the photocatalytic activity of all Fe-MCNC samples was significantly higher than that of MCN. However, as the Fe concentration grew above 2Fe-MCNC, the photocatalytic activity dropped. According to BET, the excess Fe elements tend to self-aggregate, reducing the surface area. The recycling stability of a specific photocatalyst is an important parameter for determining its applicability. Thus, the stability of the 2Fe-MCNC sample was tested three times in a row under identical conditions. As demonstrated in Figure 9c, there was no discernible deactivation in H_2_ evolution after three cycles, indicating that the 2Fe-MCNC nanostructures remained stable. Such significant enhancement is caused by the combined effects of Fe-doping, supramolecular approaches, and thermal exploration. The standard deviation (SD) of reproducibility in our study of the MCN-1H and 2Fe-MCNC catalysts was determined, and the values are shown in Table 3 (here, we have only shown the reproducibility data for the 2Fe-MCNC catalyst).

The supramolecular approach, a second time of thermal exfoliation of the synthesized samples, and the doping of iron all contributed to the exfoliation’s encouragement and, ultimately, to the expansion of the MCNC’s surface area. Higher specific surfaces have the ability to generate more electron-hole pairs, absorb more light, and accelerate the in-plane electron transport. These two advantages eventually improved the photocatalytic performance. The Fe ion produced a new impurity band when it was coordinated with the aromatic rings [51]. This impurity band served as both a bridge and barrier, enhancing the separation of the photogenerated electrons and holes. Furthermore, the coordination effect between Fe and the aromatic rings reduced the band gap and enhanced the visible light absorption zones, meaning that more photo-induced carriers were formed under the same light irradiation conditions. When the concentration of Fe^3+^ reaches a certain level, the surplus Fe species may act as recombination centers for photoinduced electrons and holes [19]. Furthermore, high Fe levels may break the MCN structure, resulting in a decrease in the specific surface of the NPs, as clearly observed in the FE-SEM images of 3Fe-MCNC 9as seen in Figure 1g–h.

**Figure 10:**
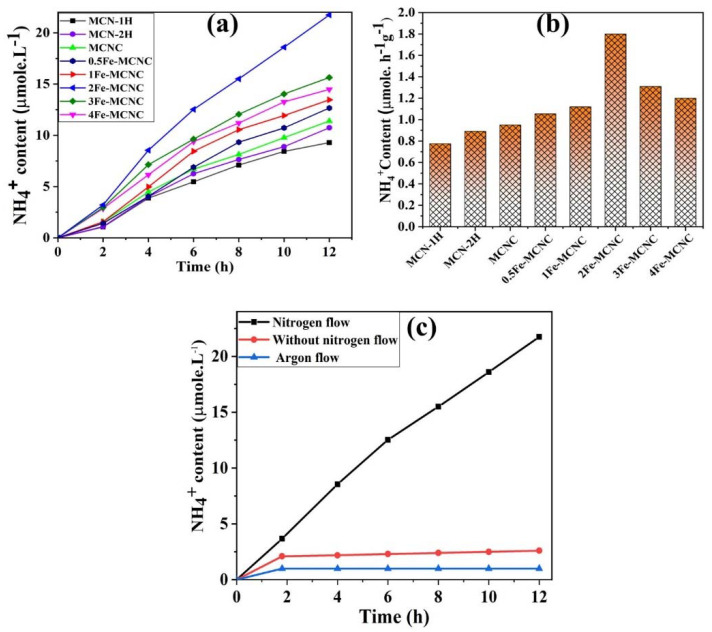
(**a**,**b**) The photocatalytic N_2_ fixation performance of MCN and the Fe-MCNC nanostructure. (**c**) Control experiment for photocatalytic N_2_ fixation.

Photocatalytic nitrogen fixation was performed under 65 W LED (R51060-TERTEC) white light irradiation to evaluate the photocatalytic properties of the synthesized catalysts. The photocatalytic nitrogen fixation over MCN and Fe-MCNC is shown in Figure 10, under white illumination, where Fe-MCNC improved the photocatalytic performance for nitrogen fixation, and 2Fe-MCNC demonstrated the highest photocatalytic activity compared to MCN-1H. Excessive Fe doping increased the charge carrier recombination due to the high concentration of defects, lowering the photocatalytic efficiency [52]. As illustrated in Figure 10b, 2Fe-MCNC had the highest photocatalytic ammonia yield efficiency (1.88 µmole/h/g), which was 2.4 times that of MCN (0.77 µmole/h/g). As a result, after adding Nessler’s reagent, the solution color changed from light yellow to dark yellow as the irradiation time increased. Control studies were conducted to show that the formation of NH_3_ is caused by the reduction in N_2_. In the absence of light or a photocatalyst, no ammonia was identified. Furthermore, in the absence of a N_2_ flow and in the presence of an argon flow, photocatalytic nitrogen fixation was performed over 2Fe-MCNC under white light illumination, as shown in Figure 10c. In the absence of a N_2_ flow, a low photocatalytic ammonia yield efficiency (0.53 µmole/h) was recorded, and the amount of produced ammonia remained virtually unchanged after 2 h of light irradiation, indicating that the ammonia may be produced by the reduction of dissolved nitrogen in the solution. Besides, in the presence of an argon flow, no detectable ammonia was produced. As a consequence of the control trials, it was observed that the ammonia produced was due to photocatalytic N_2_ reduction rather than the photocatalyst or the surrounding environment.

**Figure 11:**
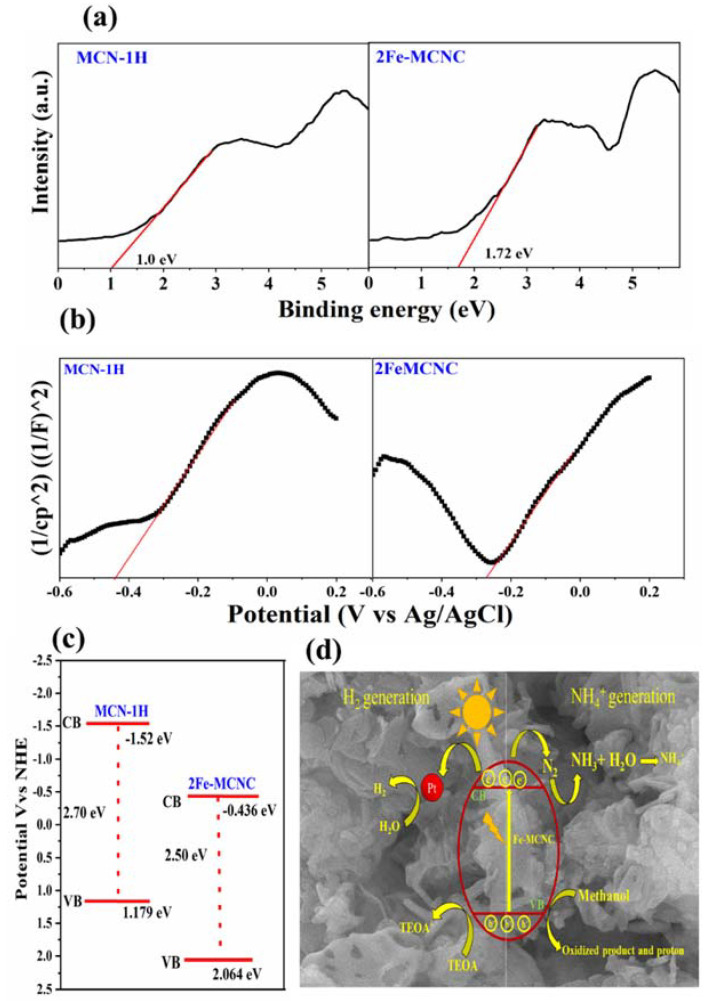
(**a**) VB XPS spectra and (**b**) the Mott–Schottky plots of MCN-1H and 2Fe-MCNC (**c**) The band structure of MCN-1H and 2Fe-MCNC; (**d**) the possible photocatalytic hydrogen generation and N_2_ fixation mechanism for the Fe-MCNC nanostructure.

The VB XPS spectra were obtained to further study the influence of Fe doping on the relative locations of CB and VB, as shown in Figure 11a. When compared to the MCN spectrum, there is a clear shift (0.72eV) in 2Fe-MCNC, which can be attributed to the Fe atoms doped into the MCN lattice. With the help of the VB XPS spectra, a Mott–Schottky plot, as shown in Figure 11b, and the band gap from the UV DRS study were used to determine the VB and CB positions of representative samples, as shown in Figure 11c. The VB of MCN and 2Fe-MCNC were located at +1.119 and +2.064eV, respectively. Combined with the UV-Vis result, the optical CB potentials of MCN and 2Fe-MCNC were located at −1.56 and −0.43 eV, respectively. Evidently, the CB and VB positions gradually altered with an increase in Fe doping. Such tunable CB and VB potentials are beneficial for improved photocatalytic activity [53].

Based on the study and discussion described above, an attainable mechanism for photocatalytic hydrogen evolution and ammonia production over the Fe-MCNC nanostructure was proposed, as shown in Figure 11d. For easier comprehension, the photocatalytic nitrogen fixation process is split into three steps. First, light irradiation was used to move photo-generated electrons in Fe-MCNC from the valence band to the conduction band. When electrons and holes reach the surface of photocatalysts, they will recombine synchronously. Second, methanol was used as a sacrificial reagent to trap the photogenerated holes during the photocatalytic process since holes have a high oxidation potential [54,55]. It was first oxidized, then reduced to formaldehyde and protons. Before interacting with the protons to produce ammonia, the electrons reduced the adsorbed N_2_ molecules on the active sites.

## 4. Conclusions

In conclusion, by using a supramolecular method and thermal exfoliation, we have successfully synthesized a highly effective Fe-doped MCNC nanostructure. The results of the UV-DRS study confirm the effects of the supramolecular approach and Fe doping on MCN, with the band gap shifting from 2.70 to 250 eV. The BET analysis confirms the drastic increase in surface area from 2.89 to 23.67 m^2^/g, up to an optimum level of Fe doping. Excess Fe doping in MCN reduces its surface area, due to Fe agglomeration, but it also promotes exfoliation of the stacked layers of MCN and causes them to break into small pieces, as observed in the FE-SEM images. Fe-MCNC shows effective photocatalytic activity for hydrogen evolution and nitrogen fixation, according to the photocatalytic investigations. Overall, 2Fe-MCNc is the best photocatalyst; it shows a 13.3-fold and 2.4-fold enhancement in hydrogen evolution and nitrogen fixation activity, compared to MCN-1H. Additionally, the doped Fe ion in MCNC may exist as Fe^3+^ and form Fe-N bonds with N atoms. This technique may offer a simple, suitable, practical, and useful method for the synthesis of highly effective visible light photocatalysts.

## Figures and Tables

**Table 1:** Elemental composition of the synthesized catalyst.

Samples	N	C	O	Fe
MCN-1H	53.91	43.04	3.05	0
1Fe-MCNC	51.04	47.31	1.56	0.09
2Fe-MCNC	56.15	42.28	1.30	0.19
3Fe-MCNC	56.34	42.15	1.20	0.19

**Table 2:** BET analysis results of the surface area, pore volume, and average pore diameter of the MCN and Fe-MCNC nanostructures.

Samples	BET Surface Area (m^2^ /g)	Pore Volume (cm^3^ /g)	Average Pore Diameter (nm)
MCN-1H	2.89	0.055	1.50
1Fe-MCNC	13.69	0.15	2.50
2Fe-MCNC	23.67	0.26	4.0
3Fe-MCNC	19.20	0.20	1.70

**Table 3:** Standard deviation (SD), determined for the reproducibility study of the representative catalyst.

Samples	First Cycle	Second Cycle	Third Cycle
2Fe-MCNC SD values	8820.83	8801.62	8787.42
MCN-1H SD values	572.95	561.01	553.67

## Data Availability

The data presented in this study are available on request from the corresponding author.
